# OrphanAnesthesia – anesthesia recommendations for patients suffering from rare diseases

**DOI:** 10.1186/1750-1172-9-S1-O16

**Published:** 2014-11-11

**Authors:** Tino Münster

**Affiliations:** 1 Department of Anesthesiology, Friedrich-Alexander University Erlangen-Nürnberg, 91054 Erlangen, Germany

## 

Anesthesia is becoming safer over the past decades. This process was driven by the development of new anesthetics and the establishment of modern safety concepts. On the other hand anesthetists benefit from profound pathophysiological knowledge and the competence of translating patient specific conditions into individual perioperative risk. But in case of rare diseases this concept is often lacking specific knowledge about the patient conditions and its importance for anesthesia.

OrphanAnesthesia is a common project of the Scientific Working Group of Paediatric Anaesthesia of the German Society of Anaesthesiology and Intensive Care Medicine in cooperation with the European Society for Paediatric Anaesthesia. The aim of OrphanAnesthesia is the publication of anaesthesia recommendations for patients suffering from rare diseases with the target of improving patients’ safety. When it comes to the management of rare diseases, there are only spare evidence-based facts and even far less knowledge in the anaesthetic outcome. OrphanAnesthesia would like to merge this knowledge based on scientific publications and proven experience of specialists and make it available for physicians worldwide.

All OrphanAnesthesia recommendations are standardized and need to pass a peer review process. They are being reviewed by at least one anaesthesiologist and another disease expert involved in the treatment of this group of patients. The project OrphanAnesthesia is internationally orientated. Our recommendations will be published in English on the OrphanAnesthesia website as well as in the medical journal “Anaesthesiologie und Intensivmedizin” basically. At a later time we are going to make most of the translations available to attract a larger number of readers.

The first recommendations have already been published online on http://www.orphananesthesia.eu. Authors and reviewers are from all over the world. We have received a great feedback and are convinced to be able to increase the patients’ safety. Of course we also would like to increase the number of recommendations, considering the facts that there are about 8,000 diagnosed rare diseases.

